# *MYORG*-related disease is associated with central pontine calcifications and atypical parkinsonism

**DOI:** 10.1212/NXG.0000000000000399

**Published:** 2020-02-20

**Authors:** Viorica Chelban, Miryam Carecchio, Gillian Rea, Abdalla Bowirrat, Salman Kirmani, Luca Magistrelli, Stephanie Efthymiou, Lucia Schottlaender, Jana Vandrovcova, Vincenzo Salpietro, Ettore Salsano, Davide Pareyson, Luisa Chiapparini, Farida Jan, Shahnaz Ibrahim, Fatima Khan, Zul Qarnain, Stanislav Groppa, Nin Bajaj, Bettina Balint, Kailash P. Bhatia, Andrew Lees, Patrick J. Morrison, Nicholas W. Wood, Barbara Garavaglia, Henry Houlden

**Affiliations:** From the Department of Neuromuscular Diseases (V.C., S.E., L.S., J.V., V.S., N.W.W., H.H.), UCL Queen Square Institute of Neurology; National Hospital for Neurology and Neurosurgery (V.C., S.E., L.S., J.V., V.S., N.W.W., H.H.), Queen Square, London, UK; Department of Neurology and Neurosurgery (V.C., S.G.), Institute of Emergency Medicine, Chisinau, Republic of Moldova; Department of Neuroscience (M.C.), University of Padua, Italy; Northern Ireland Regional Genetics Service (G.R., P.J.M.), Belfast City Hospital, UK; Department of Neuroscience (A.B.), Interdisciplinary Center (IDC) Herzliya, Israel; Department of Paediatrics & Child Health (S.K., F.J., S.I., F.K., Z.Q.), Aga Khan University, Karachi, Pakistan; Department of Neurology (L.M.), Eastern Piedmont University, Novara, Italy; Department of Neurology (E.S., D.P.) and Department of Neuroradiology (L.C.), Fondazione IRCCS Istituto Neurologico Carlo Besta, Milan, Italy; Department of Clinical Neurology (N.B.), University of Nottingham, UK; Department of Clinical and Movement Neurosciences (B.B., K.P.B., N.W.W.), UCL Queen Square Institute of Neurology, London, UK; Department of Neurology (B.B.), Heidelberg University Hospital, Germany; Reta Lila Weston Institute (A.L.), UCL Queen Square Institute of Neurology, London, UK; and Medical Genetics and Neurogenetics Unit (B.G.), Fondazione IRCCS Istituto Neurologico Carlo Besta, Milan, Italy.

## Abstract

**Objective:**

To identify the phenotypic, neuroimaging, and genotype-phenotype expression of *MYORG* mutations.

**Methods:**

Using next-generation sequencing, we screened 86 patients with primary familial brain calcification (PFBC) from 60 families with autosomal recessive or absent family history that were negative for mutations in *SLC20A2*, *PDGFRB*, *PDGBB*, and *XPR1*. In-depth phenotyping and neuroimaging investigations were performed in all cases reported here.

**Results:**

We identified 12 distinct deleterious *MYORG* variants in 7 of the 60 families with PFBC. Overall, biallelic *MYORG* mutations accounted for 11.6% of PFBC families in our cohort. A heterogeneous phenotypic expression was identified within and between families with a median age at onset of 56.4 years, a variable combination of parkinsonism, cerebellar signs, and cognitive decline. Psychiatric disturbances were not a prominent feature. Cognitive assessment showed impaired cognitive function in 62.5% of cases. Parkinsonism associated with vertical nuclear gaze palsy was the initial clinical presentation in 1/3 of cases and was associated with central pontine calcifications. Cerebral cortical atrophy was present in 37% of cases.

**Conclusions:**

This large, multicentric study shows that biallelic *MYORG* mutations represent a significant proportion of autosomal recessive PFBC. We recommend screening *MYORG* mutations in all patients with primary brain calcifications and autosomal recessive or negative family history, especially when presenting clinically as atypical parkinsonism and with pontine calcification on brain CT.

Primary familial brain calcification (PFBC) is a genetic neurodegenerative condition characterized by calcium deposition in the basal ganglia and other brain regions usually presenting with a combination of movement disorders, migraine, psychiatric, and cognitive impairment. The exact prevalence of PFBC is unknown, but population-based genomic analysis indicates that it is underestimated and underdiagnosed,^1^ with a molecular diagnosis achieved in only up to 50% of PFBC cases.^2^ The pathogenesis of PFBC involves calcium and phosphate homeostasis via mutations in *SLC20A2* (OMIM: 158378) and *XPR1* (OMIM: 605237) and endothelial integrity and function affecting the blood-brain barrier via mutations in *PDGFB* (OMIM: 190040) and *PDGFRB* (OMIM: 173410). Among these, mutations in *SLC20A2* account for approximately 45% of all autosomal dominant and de novo reported familial cases from diverse ethnicities.^3^ However, a large proportion of autosomal recessive PFBC remain undiagnosed.^4^ Recently, biallelic mutations in *MYORG* (OMIM: 618255) have been implicated in the pathogenesis of autosomal recessive PFBC in families of Chinese^5^ and French^6^ ethnicity. Here, we report a large multicentric cohort of ethnically diverse patients with biallelic variants in *MYORG* and broaden the phenotypic spectrum related to *MYORG* mutations.

## Methods

### Patients

Patients with an autosomal recessive or negative family history and confirmed clinical and radiologic diagnosis of PFBC were recruited from multiple centers. Genetic testing was performed on stored blood samples of patients with unidentified etiologies of PFBC. Ethnically, the families were of British, Italian, Irish, Pakistani, and Israeli origin. Secondary causes of brain calcification were excluded in all cases. All cases were negative for other PFBC-related genes (*SLC20A2, PDGFRB, PDGBB,* and *XPR1*) and had comprehensive phenotyping performed by neurogenetics specialists.

In cases with biallelic *MYORG* variants, the results from additional investigations were retrospectively analyzed based on chart review where available: neuroimaging with CT in all reported cases (n = 8), brain MRI (n = 4), dopamine active transporter (DAT) scan (n = 2), and fluorodeoxyglucose-PET (n = 2). Cognitive impairment was assessed by formal psychometry.

### Genetic testing

DNA was extracted from peripheral blood. Whole-exome sequencing was performed in all families. An Illumina HiSeq4000 instrument (Illumina, San Diego, CA) was used to generate 100 bp paired-end reads. Alignment was performed using BWA (bio-bwa.sourceforge.net/)^7^ with GRCH38 as a reference. Variants were called using the GATK^8–11^ UnifiedGenotyper-based pipeline^8–10^ workflow. All variants were annotated using ANNOVAR^12^ and filtered using custom R scripts. Only novel or very rare variants with a minor allele frequency of <0.01 in the 1000 Genomes Project^13^ and Genome Aggregation Database (gnomAD)^14^ or known pathologic mutations were included. Variants were filtered for homozygous, compound heterozygous, highly deleterious, rare mutations segregating with the disease. Except for families 1 and 7, segregation was confirmed in all other families.

For every rare *MYORG* variant identified (ENST00000297625, GenBank transcript ID NM_020702), we determined pathogenicity and novelty. Pathogenicity was assessed using the American College of Medical Genetics and Genomics and the Association for Molecular Pathology recommendations for variant classification.^15^ Only pathogenic and likely pathogenic variants were included here. All pathogenic and likely pathogenic variants were confirmed with bidirectional Sanger sequencing. Primers are available in table e-1 (links.lww.com/NXG/A227).

### Standard protocol approvals, registrations, and patient consents

The individuals included in this study were recruited along with unaffected family members under ethics-approved research protocols (UCLH: 04/N034) with informed consent.

### Data availability

Anonymized data used for this study are available from the corresponding authors on reasonable request. A data access agreement needs to be signed.

## Results

### Genetic spectrum

**Figure 1:**
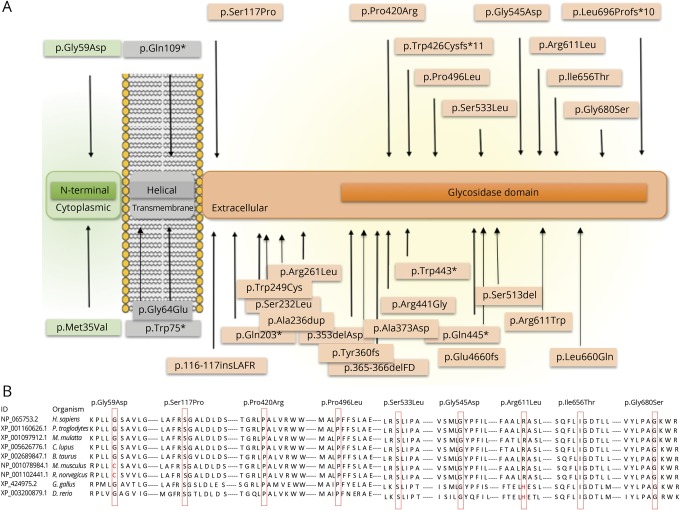
Genetic spectrum of *MYORG* mutations (A) Schematic representation of *MYORG* with all the mutations identified in our study and those reported to date. The *MYORG* functional domains and their cellular localization are indicated: green, N-terminal sequence (cytoplasmic); gray, helical (transmembrane); orange, glycosidase domain (extracellular). The mutations reported in this cohort are plotted on top of the gene; mutations previously reported are below the gene. (B) Conservation across species for novel missense *MYORG* variants. The variants are marked with red boxes for the corresponding amino acid.

We screened 86 cases from 60 families with PFBC that were negative for pathogenic variants in *SLC20A2, PDGFB, PDGFRB*, and *XPR1* and had a recessive or negative family history. We identified pathogenic and likely pathogenic homozygous and compound heterozygous variants in *MYORG* (ENST00000297625, GenBank transcript ID NM_020702) in 8 cases from 7 families (figure e-1, links.lww.com/NXG/A227). Overall, biallelic *MYORG* mutations accounted for 11.6% (7/60) of PFBC families in our cohort. We identified 12 distinct mutations, of which 4 were novel (figure 1A) and 8 were present in gnomAD with very low allele frequency in the heterozygous state and absent in the homozygous state (table e-2). With the exception of 1 variant (p.Ile656Thr), none of the variants presented here have been previously reported in *MYORG*-related brain calcifications. Apart from copy number variants, all types of mutations have been found in this cohort (1 nonsense, 1 frameshift deletion, 1 insertion, and 9 missense variants). All missense variants were located in conserved and highly conserved amino acid positions (figure 1B). The 12 mutations identified in this study were located throughout the gene with no obvious mutational hotspots. Two mutations were inherited in the homozygous state in the 2 consanguineous families; the 5 nonconsanguineous families presented with compound heterozygous variants.

### Phenotype spectrum

In our cohort, *MYORG* mutation carriers presented with a high phenotypic variability. The average age at onset was 59.1 years (median 56.4 years, range 39 years to incidental finding at 87 years). Symptoms at onset varied from parkinsonism (37.5%), ataxia and/or dysarthria (37.5%), and headache (12.5%). Insidious onset with brain calcifications found incidentally was in 12.5% of cases. Table 1 and table e-3 (links.lww.com/NXG/A227) present clinical details of all *MYORG*-related cases included in this study.

**Table 1:**
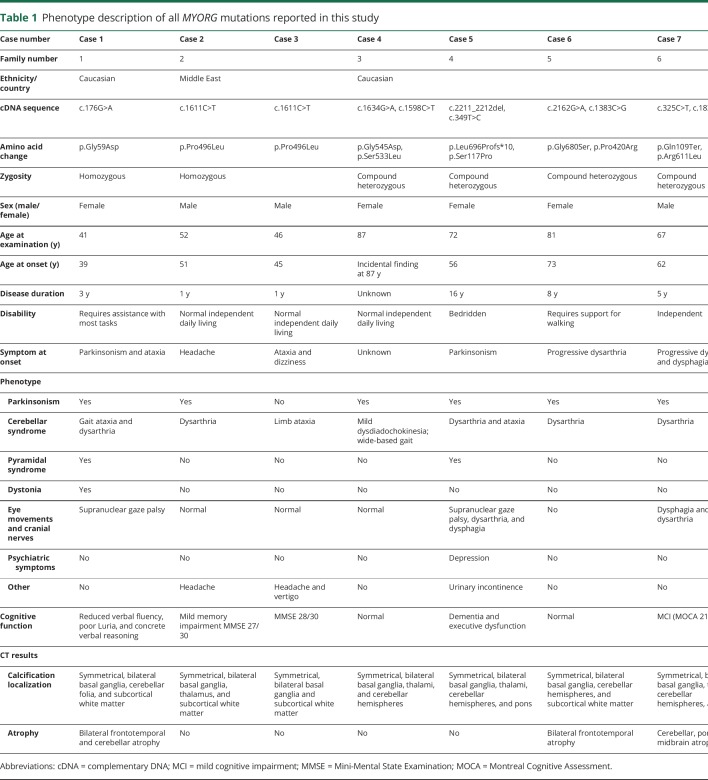
Phenotype description of all *MYORG* mutations reported in this study

An initial progressive parkinsonism associated with supranuclear gaze palsy phenotype was identified in 1/3 of cases at disease onset. Case 1 presented at the initial clinical examination age 40 years with profound facial hypomimia with a staring expression, reduced up and down gaze, associated with profound bilateral bradykinesia, rigidity, reduced arm swings, and a combination of ataxia and freezing. She had poor response to levodopa. Case 5 presented at age 56 years with asymmetric parkinsonism and supranuclear gaze palsy with poor response to levodopa. Progressive deterioration of motor function, dysarthria, dysphagia, and gait ataxia became evident over the following years. Case 8 presented at age 62 years with parkinsonism, frequent falls, staring gaze with vertical gaze palsy that progressed over 16 years along with gait ataxia, cognitive decline, urinary incontinence, and pyramidal signs. All cases presenting with parkinsonism and supranuclear gaze palsy had associated cognitive impairment characterized by executive dysfunction, poor verbal fluency, and concrete verbal reasoning with low scores on Mini-Mental State Examination (MMSE). Two of the 3 patients had a reduced tracer uptake on DAT scan consistent with symmetrical, bilateral marked loss of presynaptic dopaminergic neurons (particularly in the putamen).

A cerebellar-bulbar syndrome of variable severity was present in all our cases ranging from very mild (case 4) to moderate dysarthria and dysphagia affecting mainly speech and swallowing (cases 2, 6, and 7). Severe gait and limb ataxia was present in 3/8 of cases (cases 1, 3, and 5). Parkinsonism was detectable in 7 of 8 cases, often associated with other features including supranuclear gaze palsy, early frequent falls, early cognitive decline, and lack of response to levodopa.

One associated extrapyramidal sign in *MYORG*-related disease was limb dystonia. This was clinically presenting as dystonic posturing in the upper limb precipitated by walking. A third of our patients had bilateral pyramidal signs in the lower limbs. Other associated clinical features were headache (2 cases), urinary incontinence (2 cases), and cramps in the lower limbs (1 case).

Neuropsychiatric evaluation revealed 2 cases with depression. Cognitive assessment showed impaired cognitive function in 62.5% of cases, with different degrees of severity. *MYORG* patients showed reduced verbal fluency and poor verbal reasoning in the first year of disease (cases 1 and 3), mild memory impairment (case 2, MMSE 27/30) with progression over the following years (case 7, Montreal Cognitive Assessment 21/30 and case 8, MMSE 23/30) to a diagnosis of dementia (case 5).

Response to levodopa in cases with parkinsonian phenotype was poor to moderate and proved particularly ineffective in patients with parkinsonism associated with supranuclear gaze palsy. Case 1 with confirmed DAT scan abnormality had some modest benefit from levodopa in the first year of treatment. However, the response to treatment was short lived and faded in the next 2 years of disease.

### Neuroimaging spectrum

**Figure 2:**
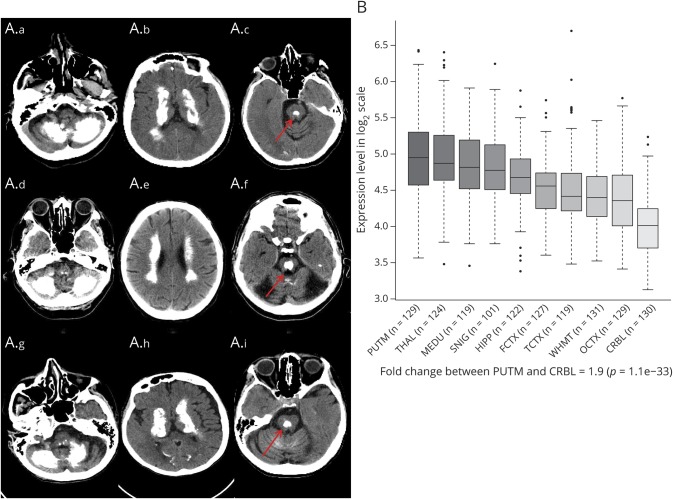
Neuroimaging spectrum in *MYORG* cases (A) Neuroimaging spectrum in *MYORG* cases. Cases 5 (A.a-A.c), 7 (A.d-A.f), and 8 (A.g-A.i) showed similar calcification pattern distribution with extensive involvement of cerebellar dentate nuclei and hemispheres, basal ganglia, thalami, and subcortical white matter; a characteristic central pontine calcification (red arrow) is present in all cases; frontotemporal and cerebellar atrophy was present in case 7; case 6: calcification of the internal globus pallidus, subcortical white matter, and dentate nuclei, with minimal involvement of thalami bilaterally. Severe frontotemporal and cerebellar atrophy is also detectable. (B) *MYORG* clinical spectrum correlates with *MYORG* gene expression in different brain areas. *MYORG* gene expression in different brain areas in adult pathologically normal human brains.^25^ *MYORG* is expressed in all 10 brain regions with highest expression detected in the putamen. CRBL = cerebellum; FCTX = frontal cortex; HIPP = hippocampus; MEDU = medulla; OCTX = occipital cortex; PUTM = putamen; SNIG = substantia nigra; TCTX = temporal cortex; THAL = thalamus; WHMT = white matter.

All patients showed extensive brain calcifications regardless of disease duration. Basal ganglia (putamen, internal globus pallidus, and caudate nucleus) were involved in all cases, whereas cerebellar hemispheres (folia and dentate nuclei) were involved in 75% of cases. Half of the cases also showed calcification of subcortical white matter. Extensive central pontine calcification was present in 3 cases. Cerebral cortical atrophy was observed in 37% of cases (figure 2A).

## Discussion

In this study, we screened *MYORG* mutations in 86 cases from 60 unrelated, autosomal recessive PFBC families. We identified 7 new families of different ethnic backgrounds with disease-causing *MYORG* variants. Biallelic *MYORG* mutations were associated with PFBCs in 11.6% of families from our cohort. We identified 12 distinct mutations, suggesting that recurrent *MYORG* mutations are infrequent. Most of the initial reported cases came from consanguineous families.^5,6^ Here, we present a cohort largely lacking in known consanguinity, with the majority of mutations inherited in the compound heterozygous state.

Our data suggest that the majority of cases have a disease onset in late adulthood with a combination of dysarthria, ataxia, parkinsonism, and cognitive decline consistent with the phenotypes previously reported in *MYORG* mutations^5,6,16–19^ and other autosomal dominant PFBC-causing genes.^20^ However, parkinsonism with supranuclear gaze palsy was frequently observed (37.8% of cases) in our cohort and has not been previously described in *MYORG* mutation carriers. Therefore, this further extends the phenotypic spectrum of *MYORG*-related disease. Of interest, central pontine calcification was present in over 1/3 of cases, which seems to be a radiologic diagnostic clue for *MYORG* mutation carriers, as this anatomic region is typically not affected in other genetic PFBC cases.^6^ As physiologic brain calcifications in this age group are reported in up to 20%,^21,22^ an association of calcifications, supranuclear gaze palsy, and parkinsonism with atypical features such as ataxia or rapid cognitive decline should prompt physicians to test for *MYORG* mutations in this subgroup of patients. We show that next-generation sequencing can contribute to the diagnosis of late-onset, mildly affected or asymptomatic cases, therefore providing a more comprehensive understanding of the genetic architecture of brain calcifications.

The exact mechanism leading to disease in *MYORG* mutations is still unknown. On a cellular level, the gene is expressed in astrocytes localized to the endoplasmic reticulum^5^ and playing a role as glycosyl hydrolase.^23^ Although gene expression (Genotype-Tissue Expression^24^) is reported highest in the basal ganglia (nucleus accumbens and caudate) after the skeletal muscle, gene expression data in BRAINEAC^25^ suggest that the putamen and the thalamus express the most *MYORG* messenger RNA followed by the medulla and the substantia nigra (figure 2B). These areas are mirrored in the clinical phenotype and calcification distribution on neuroimaging assessment in our cohort. Calcifications localized in the basal ganglia structures (100%), followed by the cerebellum in 75% of our cases, subcortical white matter (50%), and the thalamus (50%).

The phenotype observed in individuals with biallelic deleterious *MYORG* variants suggests a high variability among and within families with a disease severity ranging from insidious, incidental findings to severe, rapidly progressing disease course. Asymptomatic cases with biallelic *MYORG* mutations^5^ and heterozygous mutation carriers with punctate calcifications on the brain CT have been reported^5,6,16^). Our data together with previous reports suggest a dose-dependent phenotype based on the effect of mutations on the enzymatic activity of MYORG; however, no study has evaluated the enzymatic activity in *MYORG* mutations.

We show that biallelic *MYORG* mutations represent a significant proportion of PFBC cases without mutations in other known disease-causing genes. Here, we reported 12 distinct *MYORG* variants associated with brain calcifications and extended the phenotypic spectrum of this disease including atypical parkinsonism with pontine calcification. We recommend screening *MYORG* mutations in all patients with primary brain calcifications and autosomal recessive or negative family history.

## Glossary

DAT: dopamine active transporter
gnomAD: Genome Aggregation Database
MMSE: Mini-Mental State Examination
PFBC: primary familial brain calcification

## References

[1] Nicolas G, Charbonnier C, Campion D, Veltman JA Estimation of minimal disease prevalence from population genomic data: application to primary familial brain calcification. Am J Med Genet B Neuropsychiatr Genet 2018;177:68–74. 29152850 10.1002/ajmg.b.32605

[2] Ramos EM, Carecchio M, Lemos R, et al Primary brain calcification: an international study reporting novel variants and associated phenotypes. Eur J Hum Genet 2018;26:1462–1477. 29955172 10.1038/s41431-018-0185-4 PMC6138755

[3] Westenberger A, Klein C The genetics of primary familial brain calcifications. Curr Neurol Neurosci Rep 2014;14:490. 25212438 10.1007/s11910-014-0490-4

[4] Taglia I, Bonifati V, Mignarri A, Dotti MT, Federico A Primary familial brain calcification: update on molecular genetics. Neurol Sci 2015;36:787–794. 25686613 10.1007/s10072-015-2110-8

[5] Yao XP, Cheng X, Wang C, et al Biallelic mutations in MYORG cause autosomal recessive primary familial brain calcification. Neuron 2018;98:1116–1123.e5. 29910000 10.1016/j.neuron.2018.05.037

[6] Grangeon L, Wallon D, Charbonnier C, et al Biallelic MYORG mutation carriers exhibit primary brain calcification with a distinct phenotype. Brain 2019;142:1573–1586. 31009047 10.1093/brain/awz095

[7] Li H, Durbin R Fast and accurate long-read alignment with Burrows-Wheeler transform. Bioinformatics (Oxford, England) 2010;26:589–595. 10.1093/bioinformatics/btp698 PMC2828108 20080505

[8] McKenna A, Hanna M, Banks E, et al The Genome Analysis Toolkit: a MapReduce framework for analyzing next-generation DNA sequencing data. Genome Res 2010;20:1297–1303. 20644199 10.1101/gr.107524.110 PMC2928508

[9] Van der Auwera GA, Carneiro MO, Hartl C, et al From FastQ data to high confidence variant calls: the Genome Analysis Toolkit best practices pipeline. Curr Protoc Bioinformatics 2013;43:11.10.1–11.10.33. 25431634 10.1002/0471250953.bi1110s43 PMC4243306

[10] DePristo MA, Banks E, Poplin R, et al A framework for variation discovery and genotyping using next-generation DNA sequencing data. Nat Genet 2011;43:491–498. 21478889 10.1038/ng.806 PMC3083463

[11] Danecek P, McCarthy SA BCFtools/csq: haplotype-aware variant consequences. Bioinformatics (Oxford, England) 2017;33:2037–2039. 10.1093/bioinformatics/btx100 PMC5870570 28205675

[12] Yang H, Wang K Genomic variant annotation and prioritization with ANNOVAR and wANNOVAR. Nat Protoc 2015;10:1556–1566. 26379229 10.1038/nprot.2015.105 PMC4718734

[13] Auton A, Auton A, Brooks LD, et al A global reference for human genetic variation. Nature 2015;526:68–74. 26432245 10.1038/nature15393 PMC4750478

[14] Lek M, Karczewski KJ, Minikel EV, et al Analysis of protein-coding genetic variation in 60,706 humans. Nature 2016;536:285–291. 27535533 10.1038/nature19057 PMC5018207

[15] Richards S, Aziz N, Bale S, et al Standards and guidelines for the interpretation of sequence variants: a joint consensus recommendation of the American College of Medical Genetics and Genomics and the Association for Molecular Pathology. Genet Med 2015;17:405–424. 25741868 10.1038/gim.2015.30 PMC4544753

[16] Arkadir D, Lossos A, Rahat D, et al MYORG is associated with recessive primary familial brain calcification. Ann Clin Transl Neurol 2019;6:106–113. 30656188 10.1002/acn3.684 PMC6331209

[17] Chen Y, Fu F, Chen S, et al Evaluation of MYORG mutations as a novel cause of primary familial brain calcification. Mov Disord 2019;34:291–297. 30589467 10.1002/mds.27582

[18] Forouhideh Y, Müller K, Ruf W, et al A biallelic mutation links MYORG to autosomal-recessive primary familial brain calcification. Brain 2019;142:e4. 30649222 10.1093/brain/awy343

[19] Peng Y, Wang P, Chen Z, Jiang H A novel mutation in MYORG causes primary familial brain calcification with central neuropathic pain. Clin Genet 2019;95:433–435. 30460687 10.1111/cge.13467

[20] Batla A, Tai XY, Schottlaender L, Erro R, Balint B, Bhatia KP Deconstructing Fahr's disease/syndrome of brain calcification in the era of new genes. Parkinsonism Relat Disord 2017;37:1–10. 28162874 10.1016/j.parkreldis.2016.12.024

[21] König P Psychopathological alterations in cases of symmetrical basal ganglia sclerosis. Biol Psychiatry 1989;25:459–468. 2930811 10.1016/0006-3223(89)90199-6

[22] Yamada M, Asano T, Okamoto K, et al High frequency of calcification in basal ganglia on brain computed tomography images in Japanese older adults. Geriatr Gerontol Int 2013;13:706–710. 23279700 10.1111/ggi.12004

[23] Datta K, Guan T, Gerace L NET37, a nuclear envelope transmembrane protein with glycosidase homology, is involved in myoblast differentiation. J Biol Chem 2009;284:29666–29676. 19706595 10.1074/jbc.M109.034041 PMC2785598

[24] GTEx Consortium. Human genomics. The genotype-tissue expression (GTEx) pilot analysis: multitissue gene regulation in humans. Science 2015;348:648–660. 25954001 10.1126/science.1262110 PMC4547484

[25] Ramasamy A, Trabzuni D, Guelfi S, et al Genetic variability in the regulation of gene expression in ten regions of the human brain. Nat Neurosci 2014;17:1418–1428. 25174004 10.1038/nn.3801 PMC4208299

